# Mithramycin A suppresses basal triple-negative breast cancer cell survival partially via down-regulating Krüppel-like factor 5 transcription by Sp1

**DOI:** 10.1038/s41598-018-19489-6

**Published:** 2018-01-18

**Authors:** Rong Liu, Xu Zhi, Zhongmei Zhou, Hailin Zhang, Runxiang Yang, Tianning Zou, Ceshi Chen

**Affiliations:** 1 Kunming Institute of Zoology, Chinese Academy of Sciences, Key Laboratory of Animal Models and Human Disease Mechanisms of Chinese Academy of Sciences & Yunnan Province, Kunming, 650223 China; 2 Center for Reproductive Medicine, Department of Obstetrics and Gynecology, Peking University Third Hospital, Beijing, 100191 China; 3 Department of Oncology, Yunnan Tumor Hospital, The Third Affiliated Hospital of Kunming Medical University, Kunming, Yunnan 650118 China; 4 Department of Breast Surgery, Yunnan Tumor Hospital, The Third Affiliated Hospital of Kunming Medical University, Kunming, Yunnan 650118 China

## Abstract

As the most malignant breast cancer subtype, triple-negative breast cancer (TNBC) does not have effective targeted therapies clinically to date. As a selective Sp1 inhibitor, Mithramycin A (MIT) has been reported to have anti-tumor activities in multiple cancers. However, the efficacy and the mechanism of MIT in breast cancer, especially TNBC, have not been studied. In this study, we demonstrated that MIT suppressed breast cancer cell survival in a dosage-dependent manner. Interestingly, TNBC cells were more sensitive to MIT than non-TNBC cells. MIT inhibited TNBC cell proliferation and promoted apoptosis *in vitro* in time- and dosage-dependent manners. MIT suppressed TNBC cell survival, at least partially, by transcriptionally down-regulating KLF5, an oncogenic transcription factor specifically expressed in basal TNBC. Finally, MIT suppressed TNBC cell growth in a xenograft mouse model. Taken together, our findings suggested that MIT inhibits basal TNBC via the Sp1/KLF5 axis and that MIT may be used for TNBC treatment.

## Introduction

Breast cancer is the most commonly diagnosed malignancy among women in China, accounting for approximately 15% of new cancers in women^1^. Breast cancer is highly heterogeneous and is clinically classified into several subtypes based on the expression of the estrogen receptor (ERα), PR (progesterone receptor), and human epidermal growth factor 2 (HER2): the luminal A subtype (ERα+ and/or PR+ with low levels of Ki-67), the luminal B subtype (ERα+ and/or PR+ with high levels of Ki-67), the HER2 subtype (HER2+), and triple-negative subtype (ER−/PR−/HER2−, TNBC)^2,3^. Compared to other subtypes, TNBC is associated with higher cancer grades, higher metastasis rates, and poorer prognosis^4,5^. Furthermore, TNBC with high Ki-67 levels and BRCA gene mutations have been reported to have poorer prognosis^6–8^. For women with metastatic TNBC, the 5-year survival rate is less than 30%^4^. Furthermore, there are no effective targeted therapies for TNBC, and adjuvant chemotherapy is currently the only treatment option clinically. Although some molecules, such as poly-ADP ribose polymerase (PARP)^9^, epidermal growth factor recptor (EGFR)^10^, phosphoinositide 3-kinase (PI3K)/AKT^11^, etc. have been revealed to be promising molecular targets for TNBC treatment, and their inhibitors have now entered clinical trials, no targeted therapy has been approved for TNBC patients currently. Therefore, there is an urgent need to develop effective treatments for this aggressive subtype of breast cancer. TNBC is also heterogeneous and may be further classified into 7 subtypes based on gene expression profiling^12^.

As a member of the Krüppel-like transcription factor family, human Krüppel-like factor 5 (KLF5) has been implicated in promoting breast cell proliferation, survival, migration, stemness maintenance, and tumorigenesis^13–16^. High KLF5 expression is an unfavorable prognostic marker correlated with shorter survival for breast cancer patients^17,18^. We previously found that KLF5 is highly and specifically expressed in basal TNBC cell lines^19^, and depletion of KLF5 significantly suppresses basal TNBC cell proliferation, survival, and tumor growth^20–22^. Pharmacological inhibition of KLF5 suppressed TNBC stemness and growth^16,23^. Taken together, KLF5 could serve as a promising therapeutic target for basal TNBC.

Specificity protein 1 (Sp1), a member of the Sp/Krüppel-like factor transcription factor family, regulates multiple cellular functions and promotes tumor progression by regulating cell cycle, apoptosis, and metastasis^24–26^, via binding to GC-rich sequences of the target genes’ promoter regions^27^. Inhibition of Sp1 has been found to suppress cell survival in various cancer cell models^28,29^. We previously reported that Sp1 is essential for basic transcription of *KLF5*^30^. As a selective Sp1 inhibitor, Mithramycin A (MIT) inhibits Sp1 binding to DNA. It has been reported that MIT has anti-tumor activities in multiple cancers, including prostate cancer^31^, cervical cancer^32^, lung and esophageal cancer^33^, etc. Furthermore, MIT has been clinically used to treat several types of cancer, including testicular cancer^34^ and chronic myeloid leukemia^35^. However, whether MIT has anti-tumor effects on breast cancer, especially TNBC, has not been elucidated.

In the present study, we demonstrated that MIT suppressed breast cancer cell survival, especially TNBC cell survival. MIT suppressed *KLF5* transcription in time and dosage-dependent manners via inhibiting Sp1 binding to the *KLF5* promoter. Ectopic overexpression of KLF5 partially rescued MIT-induced loss of cell viability. Our data suggest that MIT could serve as a candidate therapeutic drug for basal TNBC.

## Results

### MIT strongly suppresses KLF5-positive TNBC cell growth

**Figure 1:**
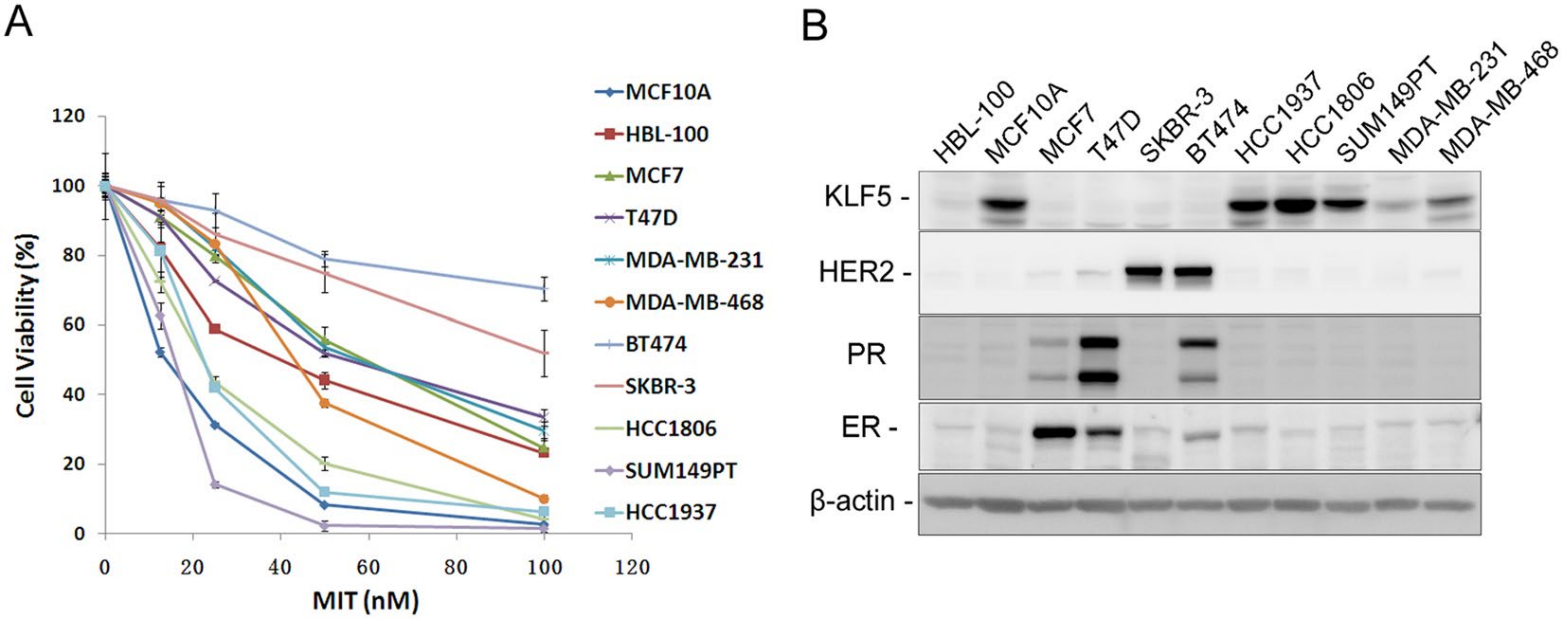
KLF5 expression levels are positively correlated with MIT sensitivity in breast cell lines (**A**). MIT decreased cell viability of breast cell lines. Eleven breast cell lines were plated in 48-well plates at a density of 1 × 10^4^ − 4 × 10^4^/well. One day after cell seeding, the cells were treated with MIT or DMSO control at indicated dosages for another 48 hours. The cells were then fixed for the SRB assay to measure cell viability. (**B**) The protein expression of KLF5, ERα, PR and HER2 in 11 breast cell lines was measured by immunoblotting. β-actin was used as a loading control.

To investigate the inhibitory effects of MIT on breast cancer cells, we treated 11 breast cell lines, including immortalized breast cell lines (HBL-100 and MCF10A), ER+ breast cancer cell lines (MCF7 and T47D), HER2+ breast cancer cell lines (SKBR-3 and BT474) and TNBC cell lines (HCC1937, HCC1806, SUM149PT, MDA-MB-231 and MDA-MB-468), with MIT and measured cell viability. As shown in Fig. 1A, breast cells showed various sensitivities to MIT, with IC_50_ ranging from 12.9 nM to more than 100 nM (Table 1). Interestingly, basal triple-negative breast cell lines with a high KLF5 expression level, including MCF10A, SUM149PT, HCC1937 and HCC1806 were relatively more sensitive to MIT than other cell lines (Fig. 1B).

**Table 1:** Effects of MIT on cell viability of breast cell lines.

Cell lines	IC_50_ (nM)
MCF10A	12.9
HBL-100	39.5
MCF7	58.4
T47D	56.5
BT474	>100
SKBR-3	>100
HCC1937	22.4
HCC1806	22.2
SUM149PT	15.3
MDA-MB-231	56.6
MDA-MB-468	42.4

### MIT suppresses cell proliferation and induces apoptosis

**Figure 2:**
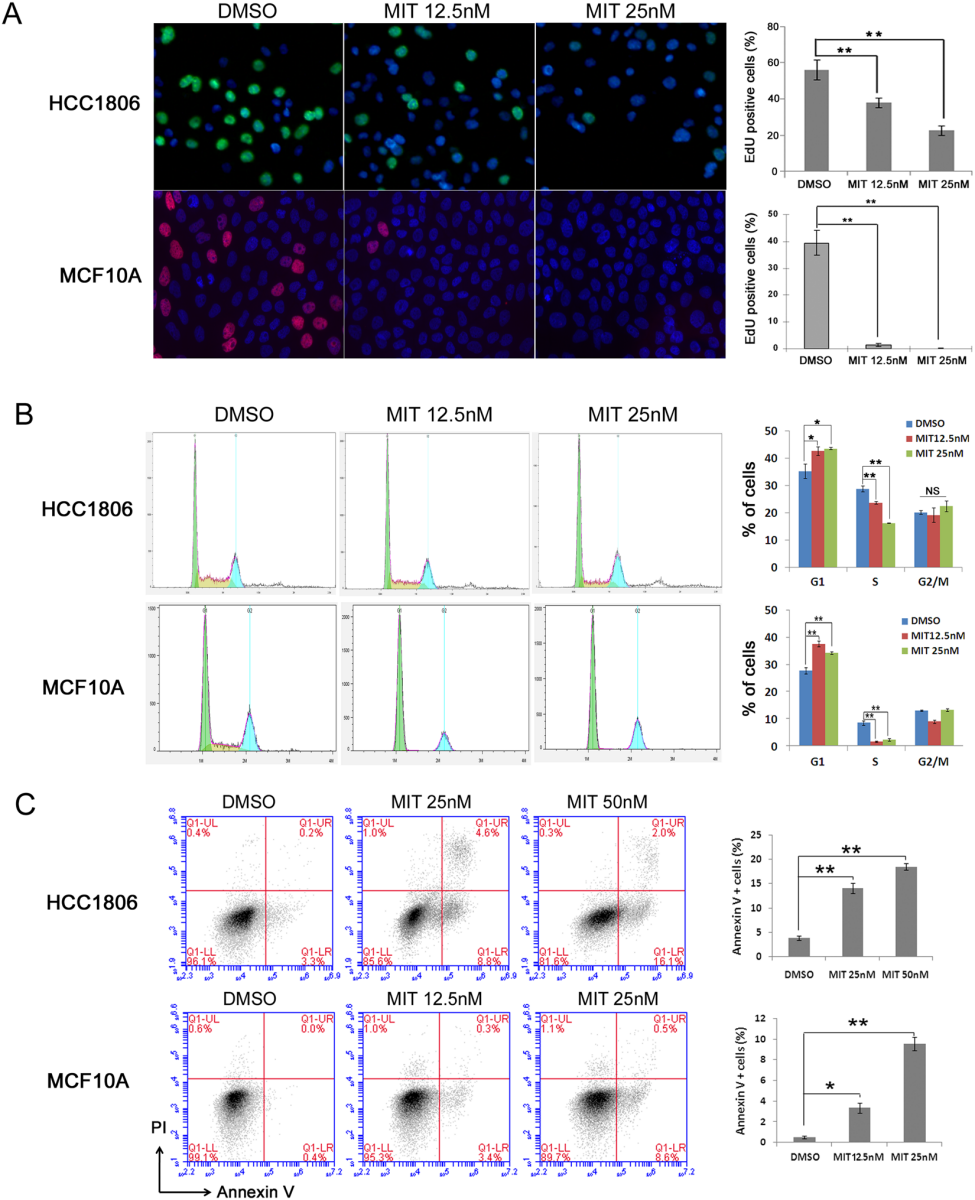
MIT inhibits DNA synthesis, G1/S cell cycle progression, and survival (**A**). MIT suppressed HCC1806 and MCF10A cell proliferation in a dosage-dependent manner. MIT significantly inhibited DNA synthesis, as examined using the Click-iT^TM^ EdU Alexa Fluor^®^ 488/647 Imaging Kit. The quantitative results are shown on the right. (**B**) MIT inhibited G1/S cell cycle progression in a dosage-dependent manner. HCC1806 and MCF10A cells were plated and treated with MIT or DMSO for 24 hours. The cells were then collected and fixed for cell cycle analysis. (**C**) MIT induced HCC1806 and MCF10A cell apoptosis in a dosage-dependent manner. HCC1806 and MCF10A cells were plated and treated with MIT or DMSO at indicated concentrations for 48 and 24 hours, respectively. The cells were then collected for Annexin V staining and flow cytometry analysis. *P < 0.05, **P < 0.01, t-test.

Since MIT significantly decreased cell viability (Fig. 1), we first analyzed cell proliferation of HCC1806 and MCF10A after MIT treatment using the EdU-incorporation assay. As shown in Fig. 2A, MIT significantly inhibited DNA synthesis in a dosage-dependent manner in both cell lines. Moreover, we examined the cell cycle and found that MIT significantly inhibited G1/S cell cycle progression in a dosage-dependent manner (Fig. 2B). Apoptosis was analyzed by Annexin V staining and flow cytometry analysis. MIT significantly increased Annexin V positive apoptotic cells in both HCC1806 and MCF10A cells in a dosage-dependent manner (Fig. 2C).

### MIT down-regulates KLF5 expression

**Figure 3:**
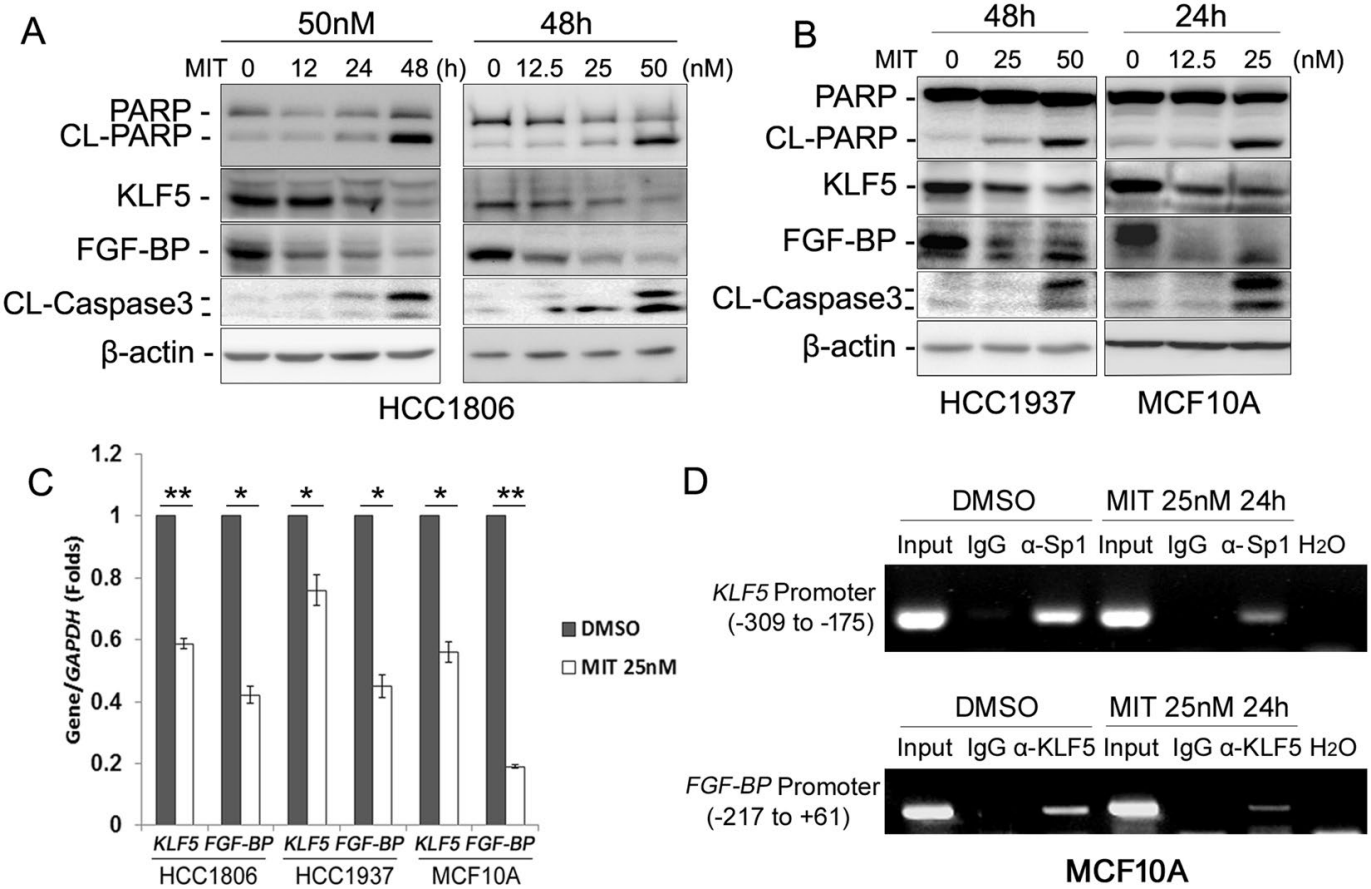
MIT down-regulates KLF5 and FGF-BP expression. (**A**) MIT down-regulated the KLF5 and FGF-BP protein levels and induced the cleavage of PARP and caspase 3 in a time- and dosage-dependent manner in HCC1806 cells. Protein levels were detected by WB. β-actin was used as the loading control. (**B**) MIT suppressed KLF5 and FGF-BP protein expression levels in HCC1937 and MCF10A cell lines. (**C**) MIT suppressed *KLF5* and *FGF-BP* expression at the mRNA level. HCC1806, HCC1937 and MCF10A cells were plated and treated with 25 nM MIT for 24 hours. The cells were collected with Trizol and total RNA was extracted for detection of *KLF5* and *FGF-BP* mRNA expression levels. (**D**) MIT suppressed Sp1 binding to the *KLF5* promoter and KLF5 binding to *FGF-BP* promoter as determined by ChIP assays. The input DNA and water were used as positive and negative controls, respectively.

Our previous studies showed that KLF5 is a key oncogenic transcription factor in basal TNBC cells^20^, and Sp1 is a key transcription factor for *KLF5* transcription^30^. MIT is well known to inhibit Sp1 recruitment to its target gene promoters^36,37^. We hypothesize that MIT suppresses basal TNBC through down-regulating the KLF5 expression by blocking the recruitment of Sp1 to the *KLF5* gene promoter. To test this hypothesis, we treated HCC1806 cells with MIT and detected KLF5 expression. As shown in Fig. 3A, MIT suppressed KLF5 and its downstream target gene *FGF-BP* expression in a time- and dosage-dependent manner. Similar results were observed in HCC1937 and MCF10A cell lines (Fig. 3B). Consistently, MIT induced apoptosis in these cell lines as evidenced by the increase of cleaved PARP and Caspase 3 (Fig. 3A,B).

Since MIT inhibits Sp1 binding to promoters, we reasoned that MIT should inhibit the expression of KLF5 and FGF-BP at the transcriptional level. As expected, MIT significantly decreased *KLF5* and *FGF-BP* mRNA expression levels in all three cell lines examined (Fig. 3C). Using ChIP assays, we demonstrated that MIT (25 nM) suppressed Sp1 recruitment to the KLF5 promoter region. Interestingly, MIT also inhibited KLF5 recruitment to the *FGF-BP* gene promoter (Fig. 3D). These results suggested that MIT may suppress *KLF5* transcription by Sp1.

### MIT decreases cell viability partially via down-regulating KLF5 expression

**Figure 4:**
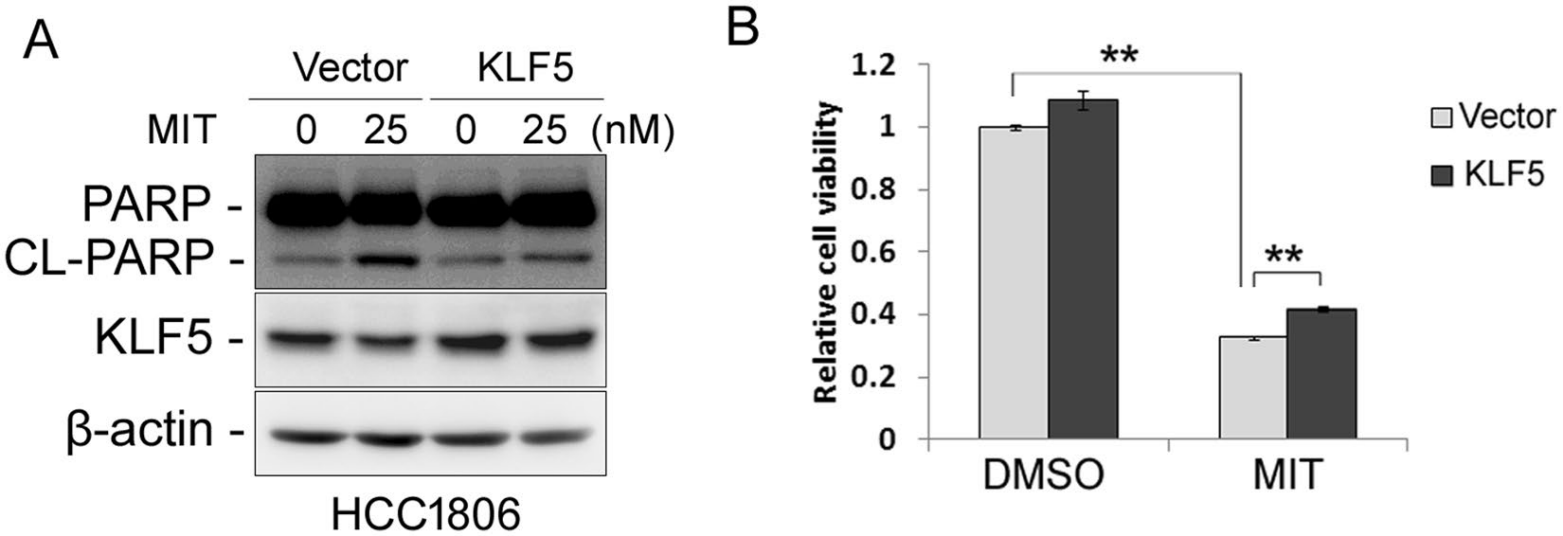
Ectopic over-expression of KLF5 partially rescues MIT-induced apoptosis and cell viability reduction in HCC1806. (**A**) KLF5 over-expression decreases MIT-induced PARP cleavage in HCC1806 cells. HCC1806 cells were infected with FUCGW-KLF5 or vector control and treated with 25 nM MIF for 24 h. The apoptosis marker cl-PARP was detected by WB. (**B**) Ectopic expression of KLF5 in HCC1806 cells partially rescued the MIT-induced cell viability reduction. HCC1806 cells were infected with FUCGW-KLF5 or vector control and treated with 25 nM MIF for 48 h before the cells were fixed for SRB assays. **P < 0.01, t-test.

Since MIT suppressed KLF5 expression and decreased cell viability in TNBC, we wondered whether MIT decreased cell viability through down-regulating KLF5 expression. We overexpressed KLF5 in HCC1806 and treated the cells with MIT or vehicle control. Indeed, ectopic overexpression of KLF5 significantly reduced MIT-induced loss of cell viability and apoptosis indicated by PARP cleavage (Fig. 4).

### MIT suppresses TNBC cell growth *in vivo*

**Figure 5:**
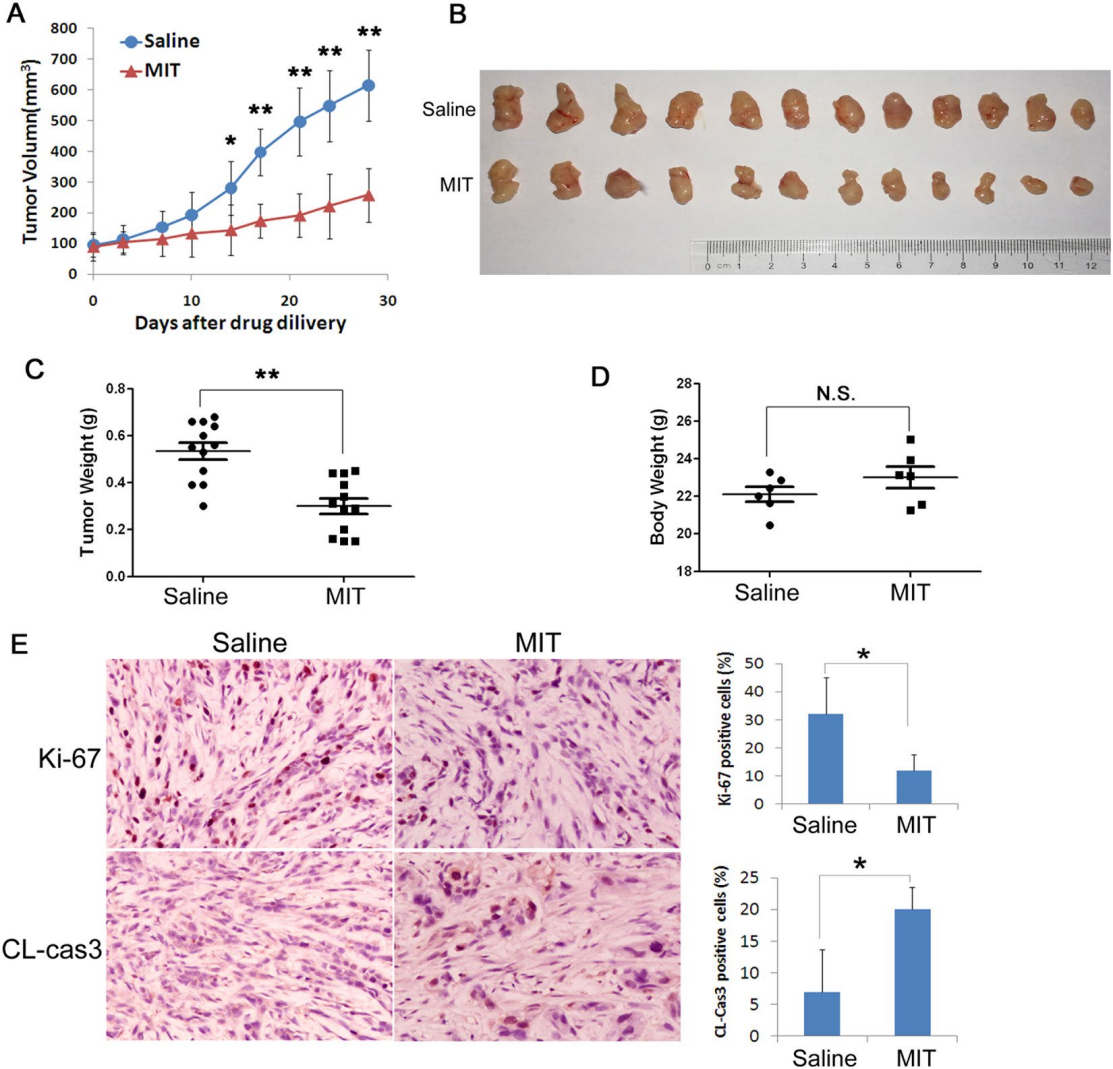
MIT suppresses HCC1806 xenograft growth and induces apoptosis *in vivo*. (**A**,**B**) MIT suppressed HCC1806 tumor growth in Balb/c nude mice. HCC1806 cells were injected into the fat pat of female Balb/c nude mice. When the average tumor size reached approximately 100 mm^3^ after inoculation, the mice were randomly and equally distributed into two groups (n = 6/group): saline control and 0.3 mg/kg MIT/d. Tumor sizes were measured twice per week for 4 weeks. Tumors were collected 4 weeks after MIT treatment. (**C**) MIT significantly decreased tumor weights compared to the saline control group (**p < 0.01, t-test). (**D**) MIT did not decrease the body weight of mice. The mice were weighed at the end of the experiment. (**E**) MIT suppressed HCC1806 cell proliferation and promoted apoptosis *in vivo*. Tumors collected from saline control and MIT groups were paraffin-fixed, sliced and stained with anti-ki-67 or cleaved-caspase 3. *p < 0.05, t-test. The quantitative results are shown on the right.

MIT suppressed TNBC cell growth and induced apoptosis *in vitro*. We then determined whether MIT suppresses tumor growth *in vivo*. We established xenografts using HCC1806 cells and treated the mice with 0.3 mg/kg MIT or saline control for 4 weeks. As expected, MIT significantly inhibited HCC1806 tumor growth (Fig. 5A,C) in nude mice without affecting the body weight of the mice (Fig. 5D). Similar results were observed in the HCC1937 xenograft model (Fig. S1A–C). Following that, we collected the tumors and detected cell proliferation by staining Ki-67 and apoptosis by staining cleaved-caspase 3 using immunohistochemistry (IHC). As shown in Fig. 5E and Figure S1D–E, MIT significantly suppressed TNBC cell proliferation and induced apoptosis *in vivo*.

## Discussion

Because TBNC is an aggressive cancer that lacks effective molecular targets for therapy, patients with TNBC typically have a relatively poorer outcome compared to those with other subtypes of breast cancer^38^. MIT showed anti-tumor activities in multiple types of cancer cells. However, whether MIT has anti-tumor effects on breast cancer cells, especially TNBC cells, has not been well elucidated. In this study, we found that MIT suppressed TNBC cell proliferation and survival *in vitro* and *in vivo*. We further demonstrated that MIT suppresses TNBC, at least partially, through down-regulating *KLF5* transcription by Sp1.

We previously reported that KLF5 promotes breast cancer cell proliferation, survival, migration, invasion and tumorigenesis^13,15,22,39^. More importantly, pharmacological inhibition of KLF5 by various inhibitors significantly suppressed cancer cell growth and/or survival. ML264, a small molecule inhibitor of KLF5, potently inhibits proliferation of colorectal cancer cells^40^. Curcumin suppresses bladder cancer cell growth by down-regulating KLF5 expression^41^. We recently reported that mifepristone^16^ and metformin^23^ inhibits KLF5 expression, cancer stem cell maintenance and tumor growth in basal TNBC. All these data suggest that KLF5 could serve as a therapeutic target for different cancers, including breast cancer. MIT efficiently suppressed TNBC cell survival *in vitro* and *in vivo*. Furthermore, ectopic over-expression of KLF5 partially but significantly rescued the MIT-induced reduction in cell viability (Fig. 4), suggesting that MIT functions, at least partially, by inhibiting KLF5 expression. Our results suggested that MIT could serve as a potential candidate for TNBC treatment, especially for TNBC patients with a high KLF5 expression level.

Previously, we have reported an Sp1-binding site between nucleotides −239 and −219 at the *KLF5* gene promoter^30^. This Sp1 site is essential for *KLF5*’s basal promoter activity and transcription. Indeed, we demonstrated that Sp1 binds to the *KLF5* promoter region containing the Sp1-binding site and MIT inhibited Sp1 binding to this site (Fig. 3). Interestingly, we noticed that MIT also inhibited KLF5 binding to its downstream target *FGF-BP* promoter (Fig. 3). Numerous studies showed that MIT binds to GC-rich DNA with a high affinity, and MIT and Sp1 family proteins, including Sp1 and KLF5, competitively bind to GC-rich motifs^36,37,42^. KLF5 is well known to bind to GC-rich sequences of downstream target gene promoters^14,39^. It is very possible that MIT also prevents KLF5 from binding the *FGF-BP* promoter.

MIT suppresses TNBC not solely by inhibiting KLF5 expression. Ectopic over-expression of KLF5 only partially rescued MIT’s anti-tumor activity in HCC1806 (Fig. 4). MIT may suppress breast cancer cell survival by regulating the expression of genes other than KLF5. Sp1 has been reported to regulate the expression of multiple genes, such as XIAP^43^, Survivin^44^, Mcl1^45^, PUMA^46^, and p21^WAF1^ ^47^. Whether these genes contribute to MIT’s functions in breast cancer cells needs further investigation.

MIT was reported to sensitize breast cancer stem cells to doxorubicin by transcriptional suppression of chemoresistant and self-renewal genes, including ABCG2, ABCC1, Bcl-2, XIAP, Oct4 and Nanog, through inhibiting Sp1 recruitment to their promoters^48^. Interestingly, KLF5 has been shown to play a crucial role in maintaining basal TNBC stem cells^16,23^.

In summary, MIT exhibited strong anti-cancer effects in breast cancer cells, especially TNBC cells, at least partially through down-regulating KLF5 expression. As a specific Sp1 inhibitor, MIT suppresses not only *KLF5* transcription by inhibiting Sp1 binding to the *KLF5* promoter but also *FGF-BP* transcription by decreasing KLF5 binding to the *FGF-BP* promoter. More importantly, MIT inhibited TNBC cell proliferation and survival in TNBC xenograft models without obvious toxic effects. Therefore, our results suggest that MIT may be a promising candidate drug for TNBC therapeutics.

## Materials and Methods

### Cell culture and drug treatment

All cell lines were purchased from the American Type Culture Collection (ATCC). The immortalized breast epithelial cell line MCF10A was maintained in DMEM/Ham’s F-1250/50 medium supplemented with 5% horse serum, 0.5 μg/ml hydrocortisone, 10 μg/ml insulin, 20 ng/ml epidermal growth factor, 0.1 μg/ml cholera enterotoxin, and 2 mM L-glutamine. TNBC cell lines HCC1806 and HCC1937 were cultured in RPMI-1640 (HyClone, Logan, UT) containing 5% fetal bovine serum (FBS), 1.5 g/L sodium bicarbonate, and 1 mM sodium pyruvate. These cells were maintained in a humidified atmosphere with 5% CO_2_ at 37 °C. Mithramycin A was purchased from Sigma-Aldrich (St. Louis, USA) and reconstituted in DMSO to a final concentration of 100 mM for storage.

### Western Blot analysis and antibodies

Western blot analysis was performed as described previously^49^. Briefly, 40 μg of each protein sample was subjected to SDS-PAGE and blotted onto polyvinylidene fluoride (PVDF) membranes. After incubating with specific primary antibodies at 4 °C overnight, membranes were incubated with horseradish peroxidase (HRP) conjugated secondary antibodies (Jackson ImmunoResearch Laboratory, West Grove, PA). The images were taken using an ImageQuant LAS4000 Biomolecular imager (GE, USA), and were processed using Adobe Photoshop Elements 2.0 (Adobe Systems Incorporated, San Jose, CA).

The anti-PARP antibody was purchased from Cell Signaling Technology (Danvers, MA). The anti-cleaved Caspase3 antibody was from Imagenex (San Diego, CA). The anti-β-actin antibody was from Sigma (St. Louis, MO). The anti-ER and anti-HER2 antibodies were from Santa Cruz (Santa Cruz, CA). The anti-PR antibody was from Invitrogen (Carlsbad, CA,). The anti-KLF5 rabbit polyclonal antibody has been described previously^49^.

### Cell proliferation and cell cycle analysis

Cell proliferation of HCC1806 and MCF10A cells was measured using the Click-iT EdU Alexa Fluor 647/488 Imaging Kit (Invitrogen), following the manufacturer’s instructions.

For cell cycle analysis, cells were plated in 6-well plates and treated with either MIT or DMSO control at an indicated dosage for 24 h before cell cycle analysis. Briefly, the cells were trypsinized and fixed with 75% ethanol at 4 °C overnight. The fixed cells were stained with propidium iodide (PI) buffer (0.3% NP-40, 0.05 mg/ml PI, 0.5 mg/ml RNase A) in the dark for 30 min at room temperature. Cell cycle was analyzed on an Accuri C6 flow cytometer (BD bioscience, San Diego, USA) within 4 h.

### Cell viability Assays

Cell viability was measured using a Sulforhodamine B assay (SRB, Sigma). Briefly, breast cells were plated in 48-well plates at densities varying from 1 × 10^4^ to 4 × 10^4^ cells/well. One day after plating, the cells were treated with either MIT or DMSO vehicle control at indicated dosage for another 48 h. The cells were then fixed with 10% trichloroacetic acid (TCA) followed by staining with 0.4% (W/V) SRB for 30 min at room temperature, and extra dye was washed with 1% acetic acid. Finally, 10 mM Tris base was added to dissolve the dye and the optical densities at 530 nm were measured using a spectrophotometric plate reader (Bio-Tek, USA).

### Overexpression of KLF5 in HCC1806

The lentiviral FUCGW-KLF5 expression vector was constructed as described previously^16^. The vector control and KLF5 expression lentiviruses were prepared according to published protocols^50^. HCC1806 cells were plated in either 12-well plates at a density of 1 × 10^5^/well or in 48-well plates at a density of 2 × 10^4^/well. Sixteen hours after plating, the cells were infected with prepared viruses. Cell culture medium was changed after 24 h. The cells were fixed for the SRB assay after treating with 25 nM MIT for 48 h.

### Tumorigenesis in nude mice

Animal care and experimental procedures were, in accordance with the Institutional Guidelines, reviewed and approved by the Animal Ethics Committee of Kunming Institute of Zoology, the Chinese Academy of Sciences, and all experiments were performed in accordance with relevant guidelines and regulations. Twelve 6-week-old female Balb/c nude mice were purchased from SJA Laboratory Animal Co., Ltd (Hunan, China). 1 × 10^6^ HCC1806 cells/point were implanted into mammary fat pads of the mice. Tumor sizes were measured using Vernier calipers once tumors became palpable. Tumor volumes were calculated using the following equation: tumor volume (cm^3^) = л(length × width^2^)/6. When the tumor size reached 100 mm^3^, the mice were randomly and equally distributed into two groups, which were treated daily with either a saline control or MIT (0.3 mg/kg). Tumor size was monitored twice per week. All mice were sacrificed and tumors were collected for analysis when the tumors reached approximately 1 cm in the largest diameter.

### Statistical analysis

All experiments were repeated at least three times. When appropriate, the data were pooled and expressed as the mean ± standard deviation and analyzed by Student’s t-test. *P* values less than 0.05 were considered as significant.

### Data availability

The datasets generated during the current study are available from the corresponding author on reasonable request.

## Electronic supplementary material

Supplementary data
