# Supplementary material for: Mithramycin A suppresses basal triple-negative breast cancer cell survival partially via down-regulating Krüppel-like factor 5 transcription by Sp1
Source: Sci Rep. 2018 Jan 18;8:1138. doi: 10.1038/s41598-018-19489-6 (PMC5773554; doi:10.1038/s41598-018-19489-6)
Supplement: Supplementary file 1 — Supplementary data [file 41598_2018_19489_MOESM1_ESM.pdf]

Mithramycin A suppresses basal triple-negative breast cancer cell survival partially via down-regulating Krüppel-like factor 5 transcription by Sp1

Rong Liu[1#], Xu Zhi[2#], Zhongmei Zhou[1], Hailin Zhang[1], Runxiang Yang[3], Tianning Zou[4], Ceshi Chen[1*]

[1]Kunming Institute of Zoology, Chinese Academy of Sciences, Key Laboratory of Animal Models and Human Disease Mechanisms of Chinese Academy of Sciences & Yunnan Province, Kunming, 650223, China

[2]Center for Reproductive Medicine, Department of Obstetrics and Gynecology, Peking University Third Hospital, Beijing 100191, China

[3]Department of Oncology, Yunnan Tumor Hospital, The Third Affiliated Hospital of Kunming Medical University, Kunming, Yunnan, 650118, China

[4]Department of Breast Surgery, Yunnan Tumor Hospital, The Third Affiliated Hospital of Kunming Medical University, Kunming, Yunnan, 650118, China

Supplementary Figure legends

Figure S1. MIT suppresses HCC1937 xenograft growth and induces apoptosis *in vivo*.

A. MIT suppressed HCC1937 tumor growth in Balb/c nude mice. HCC1937 cells were injected into the fat pat of female Balb/c nude mice. When the average tumor size reached approximately 100 mm^3 after inoculation, the mice were randomly and equally distributed into two groups (n=6/group): saline control and 0.05 or 0.15 mg/kg MIT/d. Tumor size were measured twice per week for 7 weeks. Tumors were collected 7 weeks after MIT treatment.

B. MIT significantly decreased tumor weights compared to the saline control group (**, p<0.01, t-test).

C. MIT did not decrease the body weight of mice. The mice were weighed at the end of the experiment.

D-E. MIT suppressed HCC1937 cell proliferation and promoted apoptosis *in vivo*. Tumors collected from saline control and MIT groups were paraffin-fixed, sliced and stained with anti-ki-67 (D) or cleaved-caspase 3 (E). (*, p<0.05, t-test). The quantitative results are shown on the right.

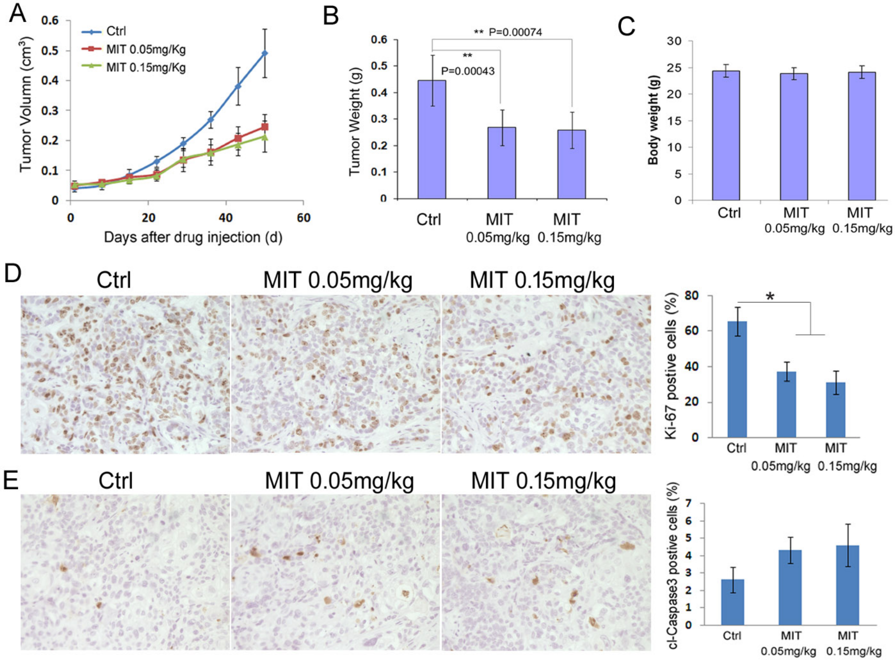
A
Ctrl
MIT 0.05mg/Kg
MIT 0.15mg/Kg
Tumor Volumn (cm³)
Days after drug injection (d)
B
** P=0.00074
**
P=0.00043
Tumor Weight (g)
Ctrl
MIT 0.05mg/kg
MIT 0.15mg/kg
C
Body weight (g)
Ctrl
MIT 0.05mg/kg
MIT 0.15mg/kg
D
Ctrl
MIT 0.05mg/kg
MIT 0.15mg/kg
*
Ki-67 postive cells (%)
E
Ctrl
MIT 0.05mg/kg
MIT 0.15mg/kg
cl-Caspase3 postive cells (%)

Figure S1
